# Takotsubo cardiomyopathy due to a pheochromocytoma: a case report

**DOI:** 10.11604/pamj.2025.51.39.47439

**Published:** 2025-06-10

**Authors:** Fatima Toulali, Afrah El Kaissi, Nada Ait Kassi, Hiba Kouira, Kaoutar Rifai, Hinde Iraqi, Mohammed El Hassan Gharbi

**Affiliations:** 1 Endocrinology Department, University Hospital of Rabat, Rabat, Morocco

**Keywords:** Atypical takotsubo, pheochromocytoma, case report

## Abstract

Takotsubo cardiomyopathy is typically triggered by severe adrenergic surges secondary to various stress factors. In rare cases, excessive catecholamine secretion due to a pheochromocytoma may be the cause. It is typically manifested by dyskinesia of the apex of the left ventricle. In this case report, we describe an atypical case of takotsubo cardiomyopathy, characterized by hypokinesia of the apical and mid-segments of the inferior wall of the left ventricle, which led to the discovery of an underlying pheochromocytoma. Although this association is rare, it is crucial to consider it, especially in atypical forms, as once the tumor is resected, the phenomenon becomes reversible.

## Introduction

Pheochromocytoma is a rare neuroendocrine tumor arising from chromaffin cells of the adrenal medulla. It leads to excessive catecholamine production, posing significant risks of morbidity and mortality, highlighting the need for prompt management [1]. Takotsubo cardiomyopathy is an uncommon manifestation of pheochromocytoma, typically characterized by transient apical myocardial dysfunction [2]. Here, we report an atypical case of takotsubo cardiomyopathy affecting the inferior wall of the left ventricle, which led to the diagnosis of an underlying pheochromocytoma.

## Patient and observation

**Patient information:** a 43-year-old female patient with a history of cholecystectomy and surgical resection of a hepatic cyst.

**Timeline:** the patient´s medical history dates back three months, with the onset of recurrent episodes of paroxysmal palpitations associated with headaches. During a consultation, hypertension was diagnosed, and the patient was started on a dual antihypertensive therapy (bisoprolol + perindopril 5 mg/5 mg). Ten days later, she presented with chest pain of anginal type, accompanied by palpitations and sweating, which led to an emergency department admission and subsequent hospitalization in cardiology for management.

**Figure 1:**
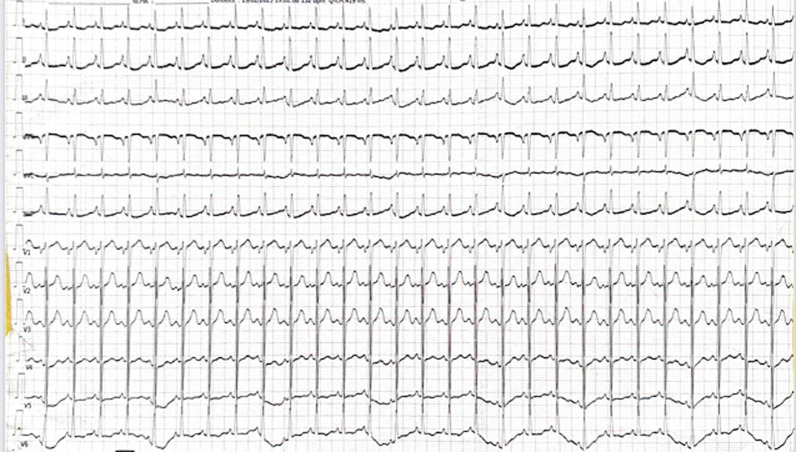
electrocardiogram at admission with anginal chest pain, revealing signs of left ventricular hypertrophy, associated with T-wave inversions in the septo-apical, high lateral, and inferior leads

**Clinical findings:** the electrocardiogram (ECG) revealed left ventricular hypertrophy (LVH) and negative T-waves in the septo-apical, high lateral, and inferior regions (Figure 1), and troponin levels were elevated.

**Figure 2:**
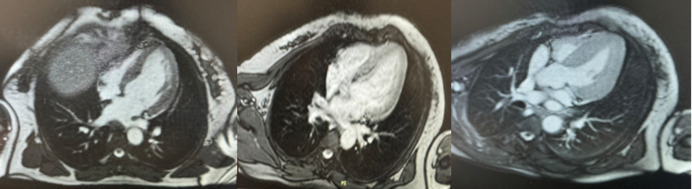
cardiac magnetic resonance imaging revealing hypokinesia of the apical and mid-segments of the inferior and inferolateral wall of the left ventricle, without evidence of infarction

**Diagnostic assessment:** the diagnosis of takotsubo cardiomyopathy was established based on the following criteria; troponin levels were elevated, coronary angiography showed no abnormalities, and the transthoracic echocardiogram (TTE) revealed a non-dilated but hypertrophied left ventricle (LV), with hypokinesia of the inferolateral wall, and a left ventricular ejection fraction (LVEF) of 62%. A complementary cardiac magnetic resonance imaging (MRI) showed hypokinesia of the apical and mid-segments of the inferior and inferolateral walls of the LV, with a LVEF of 58%, and no signs of infarction (Figure 2).

**Figure 3:**
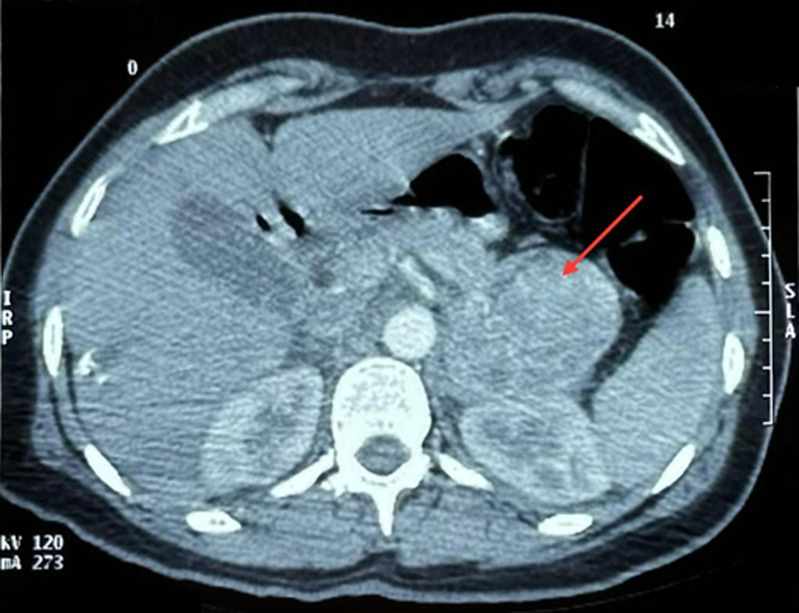
abdominal computed tomography scan revealing left adrenal mass measuring 67 x 54 x 66 mm, suggestive of pheochromocytoma

The diagnosis of pheochromocytoma was confirmed biologically by elevated plasma normetanephrine levels at 7637.6 ng/L (38 times the normal value). Morphologically, an abdominal computed tomography (CT) scan revealed a left adrenal mass with regular contours, heterogeneously enhancing early after contrast injection, measuring 67 x 54 x 66 mm, suggesting pheochromocytoma (Figure 3).

**Figure 4:**
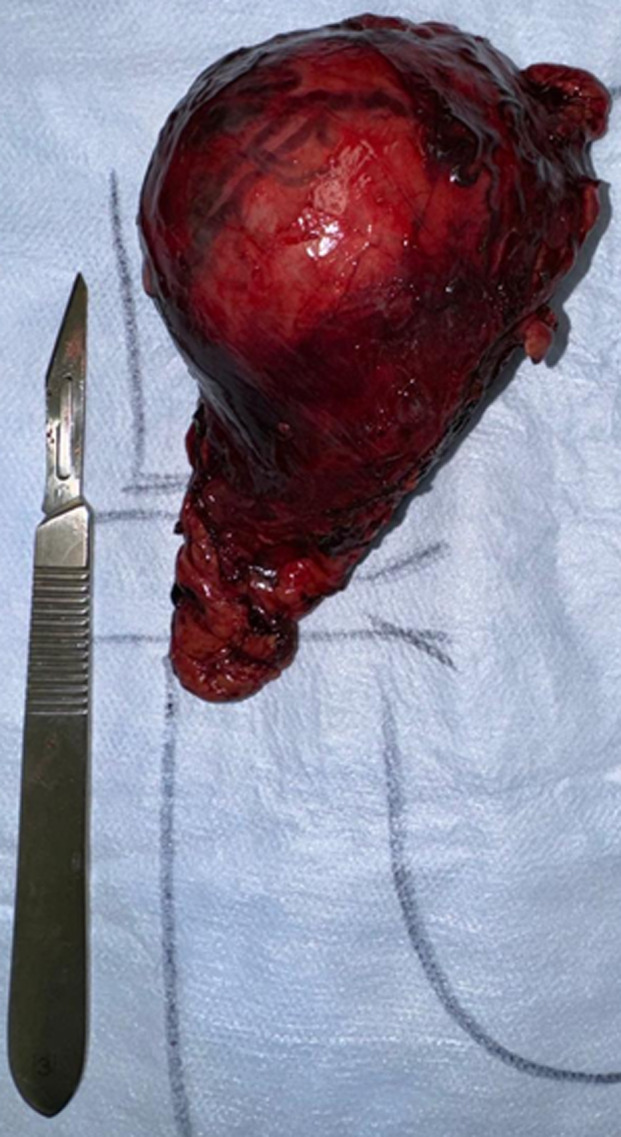
surgical specimen showing the left adrenal gland after resection

**Therapeutic intervention:** a medical preparation was initiated with an alpha-blocker gradually titrated to 6 mg/day and a beta-blocker to 110 mg/day, achieving control of both hypertension and heart rate. The patient subsequently underwent a left adrenalectomy via laparoscopy (Figure 4).

**Figure 5:**
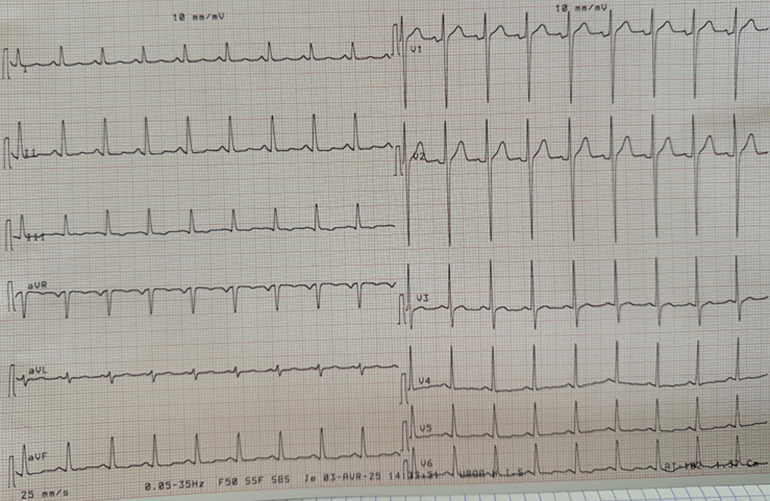
electrocardiogram after tumor removal, revealing the disappearance of signs of myocardial damage

**Follow-up and outcomes:** blood pressure and heart rate normalized after surgery, and the patient reported no further symptoms. Regarding takotsubo, follow-up TTE showed normalization of the kinetics and ventricular function. The ECG was normal (Figure 5). For the pheochromocytoma, a post-operative evaluation is planned.

**Patient perspective:** the patient provided consent for the surgical intervention and expressed satisfaction following the procedure.

**Informed consent:** written informed consent was obtained from the patient.

## Discussion

Pheochromocytoma is a rare tumor of the adrenal glands, responsible for excessive catecholamine secretion [3]. Demographically, this tumor affects adults of both sexes, typically between the ages of 30 and 50. Like many other secondary causes of hypertension, it is often underdiagnosed. A post-mortem study revealed the presence of undiagnosed pheochromocytomas in 0.05% of individuals [4]. It accounts for approximately 4% of incidental adrenal masses and is implicated in about 0.1% of hypertension cases [1].

Clinically, pheochromocytoma often presents with paroxysmal hypertension, frequently associated with headaches, tachycardia, and sweating, forming the classic Ménard triad [4]. However, it can also manifest with cardiovascular complications, including left ventricular wall dyskinesis, also known as takotsubo syndrome, first described in Japan in 1990 [3]. This syndrome typically presents with chest pain and electrocardiographic ST-segment changes mimicking an acute myocardial infarction (MI), as well as transient ballooning of the left ventricular apex, resulting from hypokinesia, akinesia, or severe dyskinesia, in the absence of epicardial coronary artery disease. This phenomenon is often accompanied by compensatory basal hyperkinesia. In general, takotsubo syndrome is benign and reversible. It is noteworthy that about 2% of patients initially diagnosed with an ST-segment elevation myocardial infarction (STEMI) actually present with takotsubo, highlighting the importance of considering this diagnosis in such cases [5,6].

The pathophysiology of takotsubo syndrome is primarily attributed to adrenergic discharge, often triggered by physical or emotional stress. However, organic causes, such as pheochromocytoma, can also be involved and should not be considered exclusion criteria for the diagnosis of this syndrome, contrary to previous suggestions [7].

In our case, takotsubo syndrome presented as severe chest pain, mimicking an acute coronary syndrome, with normal coronary angiography. The patient also exhibited paroxysmal hypertension associated with the classic Ménard triad, which suggested the diagnosis of pheochromocytoma, confirmed by elevated plasma metanephrine level. Abdominal computed tomography (CT) imaging revealed a left adrenal mass suggestive of pheochromocytoma. Regarding electrocardiographic abnormalities, these frequently include ST-segment elevation (68% of cases), primarily observed in the precordial leads, as well as diffuse T-wave inversions (97%). ST-segment depression and the presence of Q-waves are less common, occurring in 10% and 27% of cases, respectively [6]. A moderate elevation of troponins is typically observed, along with a rarer elevation of creatine kinase and its CK-MB isoenzyme, although the cardiac enzyme profile differs from that observed in MI. Indeed, cardiac enzyme levels peak early in the symptoms, without following the typical progressive course of MI. Thus, troponin and CK-MB levels are elevated in 86% and 74% of cases, respectively [6]. In our patient, the ECG showed negative T-waves in the septo-apical, high lateral, and inferior leads, and troponin was moderately elevated. Regarding left ventricular wall motion abnormalities observed in takotsubo syndrome, five variants are described: the typical form with apical ballooning, which represents 81.7% of cases, and four atypical forms: mid-ventricular (14.6%), basal or inverted (2.2%), and focal (1.5%) [8,9]. These transient wall motion abnormalities typically involve multiple vascular territories and can be detected by left ventriculography, two-dimensional echocardiography, synchronized single-photon emission tomography, and magnetic resonance imaging (MRI) [6]. The basal subtype of atypical takotsubo syndrome is a particularly rare phenotype, observed in only 2.2% of patients with this condition. It is associated with states of hypercatecholaminemia, particularly in the context of pheochromocytoma, which can account for this subtype in 30% of cases. Thus, it is strongly recommended to investigate an underlying pheochromocytoma, especially in younger patients, in whom this form is more frequently observed [3,5]. In our case, an unusual variant of takotsubo syndrome was observed, with hypokinesia of the apical and mid-segments of the inferior wall of the left ventricle, confirmed by both TTE and cardiac MRI.

In terms of prognosis, after tumor removal, rapid resolution of left ventricular dysfunction is characteristic of takotsubo syndrome and serves as a distinguishing feature compared to other forms of cardiomyopathy [5]. In our case, a rapid cardiac recovery was observed after tumor resection, with complete normalization of wall motion and ventricular function.

## Conclusion

Takotsubo syndrome induced by pheochromocytoma is rare but may progress more rapidly than other forms of this syndrome, with sometimes very severe presentations. In this context, it becomes crucial to screen for an underlying pheochromocytoma, particularly in younger patients, in complicated or recurrent cases, and when other symptoms suggestive of pheochromocytoma are present. It is also important to consider this association in atypical forms of takotsubo, which present a major diagnostic challenge due to the absence of the classic apical ballooning pattern on imaging and often nonspecific electrocardiographic changes.
